# Identification of Crucial lncRNAs for Luminal A Breast Cancer through RNA Sequencing

**DOI:** 10.1155/2022/6577942

**Published:** 2022-06-02

**Authors:** Xinjian Jia, Hai Lei, Xuemei Jiang, Ying Yi, Xue Luo, Junyan Li, Yu Chen, Sha Liu, Chengcheng Yang

**Affiliations:** Department of Breast Surgery, Deyang People's Hospital, Deyang, Sichuan Province, China

## Abstract

**Background:**

The growing body of evidence indicates aberrant expression of long noncoding RNAs (lncRNAs) in breast cancer. Nevertheless, a few studies have focused on identifying key lncRNAs for patients with luminal A breast cancer. In our study, we tried to find key lncRNAs and mRNAs in luminal A breast cancer.

**Methods:**

RNA sequencing was performed to identify differentially expressed mRNAs (DEmRNAs) and differentially expressed lncRNAs (DElncRNAs) in luminal A breast cancer. The protein-protein interaction (PPI), DElncRNA-DEmRNA coexpression, DElncRNA-nearby DEmRNA interaction networks, and functional annotation were performed to uncover the function of DEmRNAs. Online databases were used to validate the RNA sequencing result. The diagnostic value of candidate mRNAs was evaluated by receiver operating characteristic (ROC) curve analysis.

**Results:**

A total number of 1451 DEmRNAs and 272 DElncRNAs were identified. Several hub proteins were identified in the PPI network, including TUBB3, HIST2H3C, MCM2, MYOC, NEK2, LIPE, FN1, FOXJ1, S100A7, and DLK1. In the DElncRNA-DEmRNA coexpression, some hub lncRNAs were identified, including AP001528.2, LINC00968, LINC02202, TRHDE-AS1, LINC01140, AL354707.1, AC097534.1, MIR222HG, and AL662844.4. The mRNA expression result of TFF1, COL10A1, LEP, PLIN1, PGM5-AS1, and TRHDE-AD1 in the GSE98793 was consistent with the RNA sequencing result. The protein expression results of TUBB3, MCM2, MYOC, FN1, S100A7, and TFF1 were consistent with the mRNA expression result COL10A1, LEP, PLIN1, PGM5-AS1, and TRHDE-AD1 were capable of discriminating luminal A breast cancer and normal controls. Four lncRNA-nearby and coexpressed mRNA pairs of HOXC-AS3-HOXC10, AC020907.2-FXYD1, AC026461.1-MT1X, and AC132217.1-IGF2 were identified. AMPK (involved LIPE and LEP) and PPAR (involved PLIN1) were two significantly enriched pathways in luminal A breast cancer.

**Conclusion:**

This study could be helpful in unraveling the pathogenesis and providing novel therapeutic strategies for luminal A breast cancer.

## 1. Introduction

Breast cancer is the cancer with the highest impact among women [1]. Breast cancer is recognized as a malignancy with high heterogeneity. Despite better advancement in prognosis and treatment, breast cancer remains associated with major morbidity and mortality. Breast cancer can be divided into 5 “intrinsic” subtypes, including luminal A, luminal B, HER2-enriched, basal-like, and normal-like [2–6]. However, a few studies have focused on identifying diagnostic biomarkers for patients within a subtype. Therefore, identifying reliable diagnostic markers for breast cancer that distinguish between the subtypes is needed.

RNA sequencing has been increasingly used in clinical studies to define changes in gene expression [7]. In fact, the gene expression profile of RNA sequencing is often used to integrate multiple molecular events and mechanisms associated with cancer progression [8]. Long noncoding RNAs (lncRNAs), defined as transcripts greater than 200 nucleotides and nonencoding proteins, are emerging as essential regulators of mRNA expression, including transcription regulation by affecting DNA methylation or transcription factor activity [9, 10]. In addition, lncRNAs can serve as ceRNAs and participate in the regulation of encoding miRNAs [11, 12]. Accumulating number of reports have indicated that lncRNAs are associated with the majority of biological processes and diseases, including breast cancer [13]. For example, the downregulated level of NKILA is associated with breast cancer metastasis [14]. DSCAM-AS1 is an underlying treatment target that may prolong survival of luminal breast cancer patients [13]. However, research on lncRNA diagnostic biomarkers in luminal A breast cancer is rare.

In view of this, we performed RNA sequencing to identify DEmRNAs and DElncRNAs in luminal A breast cancer. The protein-protein interaction (PPI), DElncRNA-DEmRNA coexpression, DElncRNA-nearby DEmRNA interaction networks, and functional annotation were performed to uncover the function of DEmRNAs. We also performed the validation experiment and receiver operating characteristic (ROC) curve analysis. Our study identified potential key DEmRNAs and DElncRNAs in luminal A breast cancer and may provide a novel field in understanding the pathological mechanism of the disease.

## 2. Materials and Methods

### 2.1. Patients

Four patients with luminal A breast cancer were included in our study. Tumor samples and 4 paired adjacent normal tissue samples were selected from 4 patients with luminal A breast cancer. Clinical characteristics of patients are shown in Table 1. All the participants submitted the signed informed consent. The protocol was approved by the Medical Ethics Committee of Deyang People's Hospital (2017-041).

### 2.2. RNA Sequencing

Total RNA was isolated from samples using the TRIzol reagent. The RNA quality was checked using a NanoDrop ND-2000 spectrophotometer. RNA integrity was detected using agarose gel electrophoresis. Total RNA was further purified using the Ribo-Zero Magnetic kit. The Illumina HiSeq X Ten platform was used to conduct sequencing of lncRNA and mRNA. lncRNA and mRNA levels were evaluated as the sum of fragments per kilobase of the exon model per million reads mapped (FPKM). Expression levels of lncRNA and mRNA were compared using edgeR. mRNAs and lncRNAs with a *p* value <0.05 and |log2 fold change (FC)| >1 were, respectively, considered significant DEmRNAs and DElncRNAs. The heatmap of DEmRNAs and DElncRNAs in the luminal A breast cancer was obtained by using the pheatmap package in R language. In the clustering method, clustering_method parameter was set to “complete.” The method for calculating the class-clustering distance was “Euclidean.”

### 2.3. Functional Enrichment

David 6.8 was used to perform the Gene Ontology (GO) classification and Kyoto Encyclopedia of Genes and Genomes (KEGG) pathway analysis. *p* value <0.05 was considered to indicate statistically significant differences.

### 2.4. PPI Network

Top 50 upregulated or downregulated DEmRNAs in the luminal A breast cancer were used to perform the PPI network by using BioGRID and Cytoscape 3.5.1. Node and edge was used to respectively represent the protein and interaction between two proteins.

### 2.5. DEmRNA-DElncRNA Interaction Network

To identify nearby DEmRNAs of DElncRNAs with cis-regulatory effects, DEmRNAs (transcribed within a 100 kb window up- or down-stream of DElncRNAs) were searched. The DEmRNAs coexpressed with DElncRNAs were also identified. The pairwise Pearson correlation coefficients between DEmRNAs and DElncRNAs were calculated. *p* value <0.01 and the Pearson correlation coefficient |r| <0.96 were considered as significant mRNA-lncRNA coexpression pairs. The DEmRNA-DElncRNA interaction network feature was presented by Cytoscape 3.5.1.

### 2.6. Expression Validation of Identified DEmRNAs and DElncRNAs

First, the mRNA expression level of selected DEmRNAs and DElncRNAs was validated in the published Gene Expression Omnibus (GEO) dataset (GSE65194), which was published on Jan 23, 2015, and examined tissue samples consisting of 29 patients with luminal A breast cancer and 11 normal controls. Second, an online immunohistochemical study of selected DEmRNAs was conducted using “The Human Protein Atlas” database.

### 2.7. Diagnostic Analysis

To evaluate the diagnostic value of DEmRNAs and DElncRNAs in luminal A breast cancer, the “pROC” package was used to generate ROC. When the area under the ROC curve (AUC) value was greater than 0.8, the DEmRNAs/DElncRNAs were able to distinguish between case and normal controls with good specificity and sensitivity.

## 3. Results

### 3.1. DEmRNAs and DElncRNAs

**Figure 1:**
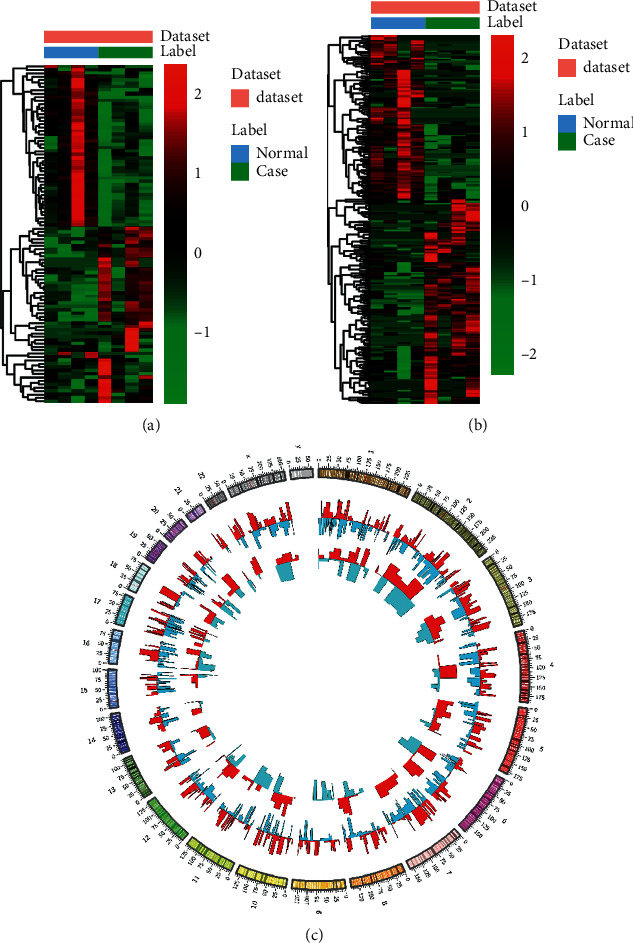
Heatmap of top 100 mRNAs and all lncRNAs in luminal A breast cancer. (a) DEmRNAs, (b) DElncRNAs, and (c) Circos plots represent the distribution of DElncRNAs and DEmRNAs on chromosomes. The outer layer cycle is the chromosome map of the human genome. The inner layers represent the distribution of DEmRNAs and DElncRNAs on different chromosomes, respectively. Red and blue colors represent upregulation and downregulation, respectively.

Based on the RNA-seq data, we found 1451 DEmRNAs and 272 DElncRNAs in the luminal A breast cancer. Among which, 651 mRNAs and 151 lncRNAs were upregulated and 800 mRNAs and 121 lncRNAs were downregulated. Top 20 mRNAs and lncRNAs are, respectively, listed in Tables 2 and 3. A heatmap of the top 100 mRNAs and all lncRNAs between the luminal A breast cancer and normal tissue is, respectively, shown in Figures 1(a) and 1(b). Circos plots represent the distribution of DElncRNAs and DEmRNAs on chromosomes (Figure 1(c)).

### 3.2. Functional Enrichment

**Figure 2:**
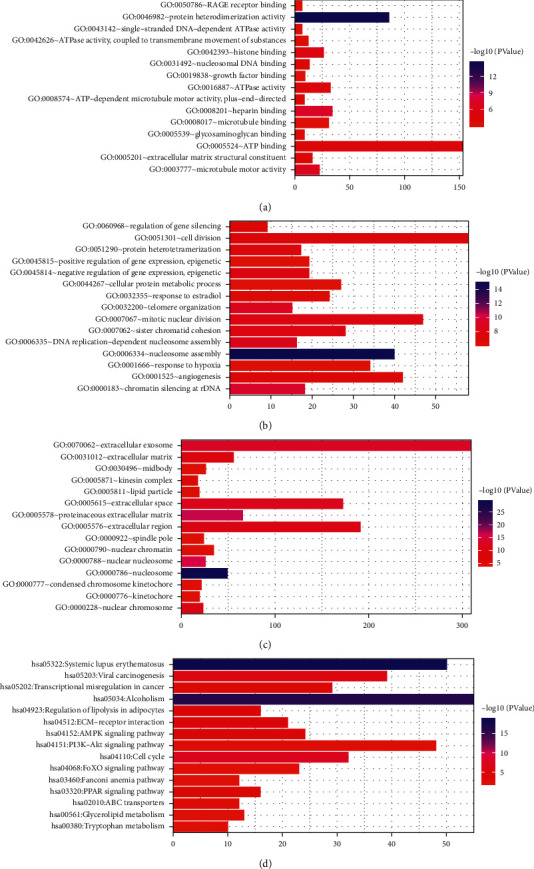
Top 15 significantly enriched GO terms and KEGG pathways in luminal A breast cancer. (a) Biological process, (b) cellular component, (c) molecular function, and (d) KEGG pathways.

GO enrichment analysis showed that these DEmRNAs were significantly enriched in the nucleosome assembly (*p* value = 1.39*E* − 15), chromatin silencing at rDNA (*p* value = 3.27*E* − 10), nucleosome (*p* value = 1.84*E* − 29), proteinaceous extracellular matrix (*p* value = 2.50*E* − 17), protein heterodimerization activity (*p* value = 4.29*E* − 15), and heparin binding (*p* value = 5.95*E* − 08). Top 15 GO terms of DEmRNAs in luminal A breast cancer are shown in Figures 2(a)–2(c). KEGG pathway enrichment analysis showed that cell cycle (*p* value = 5.85*E* − 08), viral carcinogenesis (*p* value = 6.93*E* − 06), ECM-receptor interaction (*p* value = 5.00*E* − 05), PI3K-Akt signaling pathway (*p* value = 0.001557342), and pathways in cancer (*p* value = 0.011811389) were five significantly enriched pathways in luminal A breast cancer. Top 15 significantly enriched KEGG pathways of DEmRNAs in luminal A breast cancer are shown in Figure 2(d).

### 3.3. PPI Network

**Figure 3:**
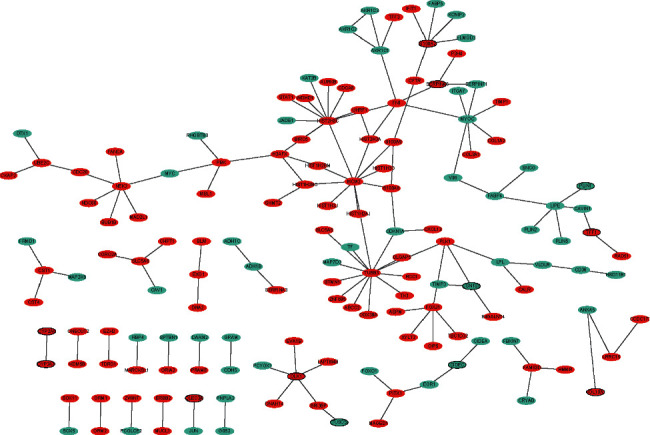
Luminal A breast cancer-specific PPI network. Ellipse and line are used to represent the node and edge, respectively. Red and green represent upward and downward adjustments, respectively. The black border indicates the top 10 upregulated and downregulated proteins.

The PPI network consisted of 150 nodes and 136 edges (Figure 3). TUBB3 (upregulation, degree = 13), HIST2H3C (upregulation, degree = 11), MCM2 (upregulation, degree = 10), MYOC (downregulation, degree = 7), NEK2 (upregulation, degree = 6), LIPE (downregulation, degree = 5), FN1 (upregulation, degree = 5), FOXJ1 (upregulation, degree = 5), S100A7 (upregulation, degree = 5), and DLK1 (upregulation, degree = 5) were considered as the hub proteins.

### 3.4. DElncRNA-Nearby DEmRNA Interaction Network

**Figure 4:**
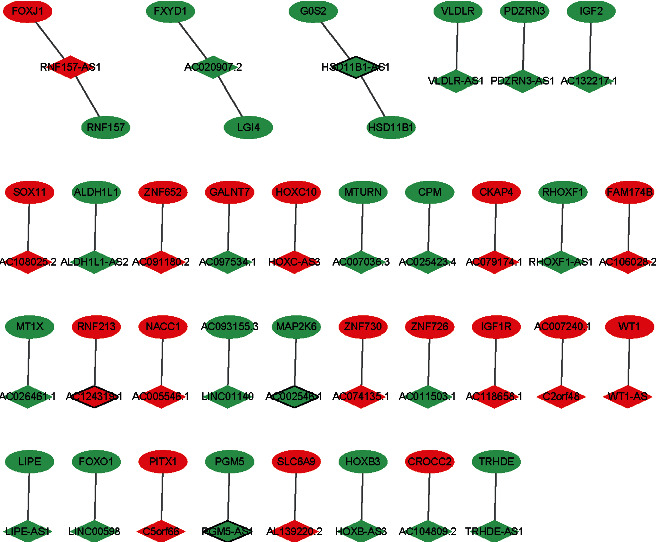
DElncRNA-nearby DEmRNA interaction network in luminal A breast cancer. Ellipse and rhombus represent DEmRNA and DElncRNA, respectively. Red and green colors represent upregulation and downregulation, respectively. The black border indicates the top 10 upregulated and downregulated lncRNAs.

In total, 37 DElncRNAs-nearby target DEmRNA pairs were identified which consisted of 34 DElncRNAs and 37 DEmRNAs (Figure 4). lncRNAs and nearby mRNAs in the DElncRNA-nearby DEmRNA pairs are displayed in Table 4.

### 3.5. DElncRNA-DEmRNA Coexpression Network

**Figure 5:**
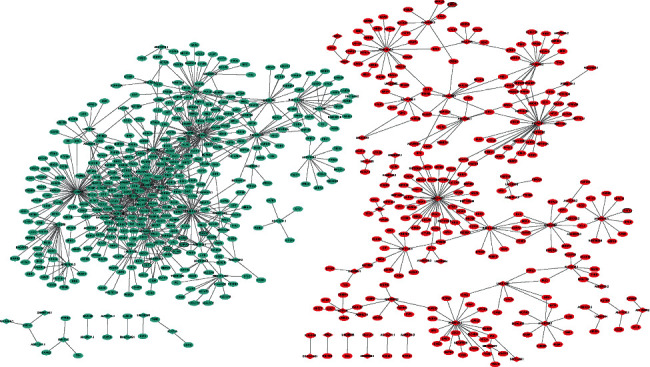
Positively coexpressed DElncRNA-DEmRNA network. Ellipse and rhombus represent DEmRNA and DElncRNA, respectively. Red and green colors represent upregulation and downregulation, respectively. The black border indicates the top 10 upregulated and downregulated DElncRNAs and DEmRNAs.

**Figure 6:**
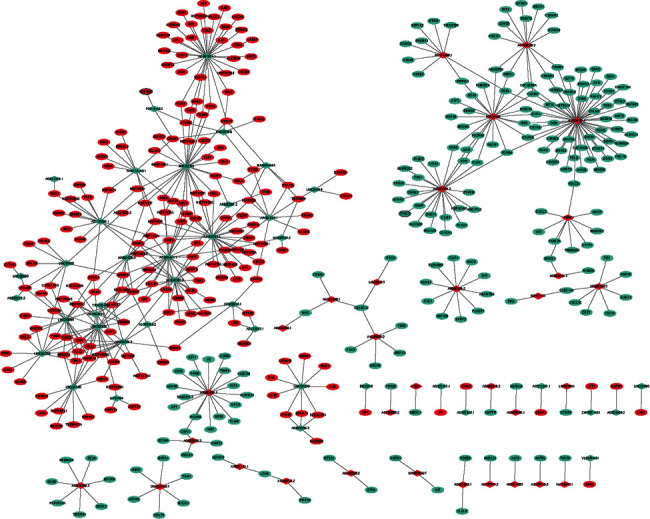
Negatively coexpressed DElncRNA-DEmRNA network. Ellipse and rhombus represent DEmRNA and DElncRNA, respectively. Red and green colors represent upregulation and downregulation, respectively. The black border indicates the top 10 upregulated and downregulated DElncRNAs and DEmRNAs.

In total, 1678 DElncRNA-DEmRNA coexpression pairs including 105 DElncRNAs and 786 DEmRNAs were identified. Among which, 1131 lncRNA-mRNA pairs were positively coexpressed (Supplementary Tables 1) and 547 lncRNA-mRNA pairs were negatively coexpressed (Supplementary Table 2). The positively coexpressed DElncRNA-DEmRNA network is shown in Figure 5. Some hub lncRNAs were identified, including AP001528.2 (downregulation, degree = 80), LINC00968 (downregulation, degree = 71), LINC02202 (downregulation, degree = 65), TRHDE-AS1 (downregulation, degree = 56), and LINC01140 (downregulation, degree = 54). The negatively coexpressed DElncRNA-DEmRNA network is shown in Figure 6. Besides being based on the relevant literature, some lncRNAs were identified according to the degree, including AL354707.1 (upregulation, degree = 54), AC097534.1 (downregulation, degree = 33), MIR222HG (downregulation, degree = 28), and AL662844.4 (downregulation, degree = 27). It is noted that a total of 4 lncRNA-mRNA pairs were identified between the DElncRNA-nearby DEmRNA interaction network and the DElncRNA-DEmRNA coexpression network, including HOXC-AS3-HOXC10, AC020907.2-FXYD1, AC026461.1-MT1X, and AC132217.1-IGF2.

### 3.6. Functional Enrichment of DEmRNAs Coexpressed with DElncRNAs

**Figure 7:**
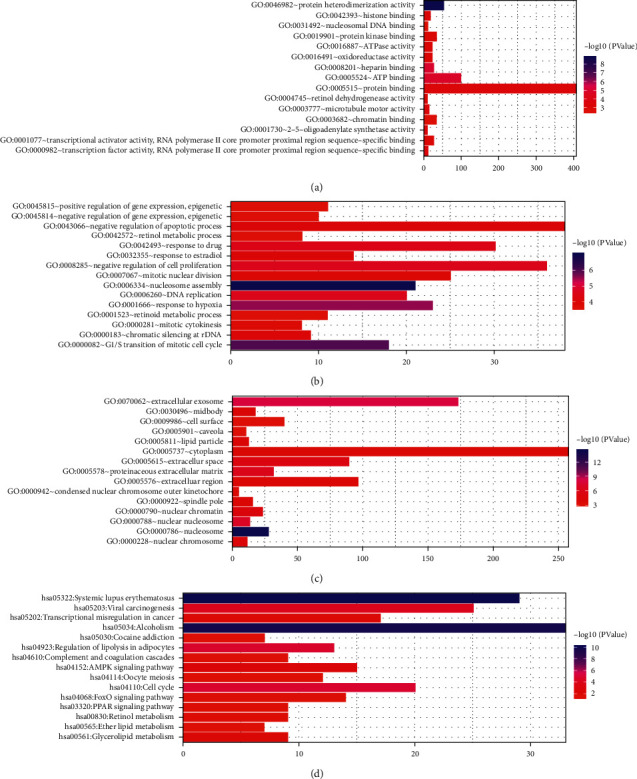
Top 15 significantly enriched GO terms and KEGG pathways of DEmRNAs coexpressed with DElncRNAs in luminal A breast cancer. (a) Biological process, (b) cellular component, (c) molecular function, and (d) KEGG pathways.

Nucleosome assembly (*p* value = 1.06*E* − 07), G1/S transition of mitotic cell cycle (*p* value = 1.08*E* − 06), nucleosome (*p* value = 3.63*E* − 15), protein heterodimerization activity (*p* value = 1.85*E* − 09), and heparin binding *p* value = 1.04*E* − 05) were most significantly enriched GO terms. Top 15 GO terms in luminal A breast cancer are shown in Figures 7(a)–7(c). In addition, we found that AMPK (involved LIPE and LEP) (*p* value = 0.003440464) and PPAR (involved PLIN1) (*p* value = 0.018732701) were two significantly enriched pathways in luminal A breast cancer. Top 15 most significantly enriched KEGG pathways of DEmRNAs in luminal A breast cancer are shown in Figure 7(d).

### 3.7. Expression Validation of Selected DEmRNAs and DElncRNAs

**Figure 8:**
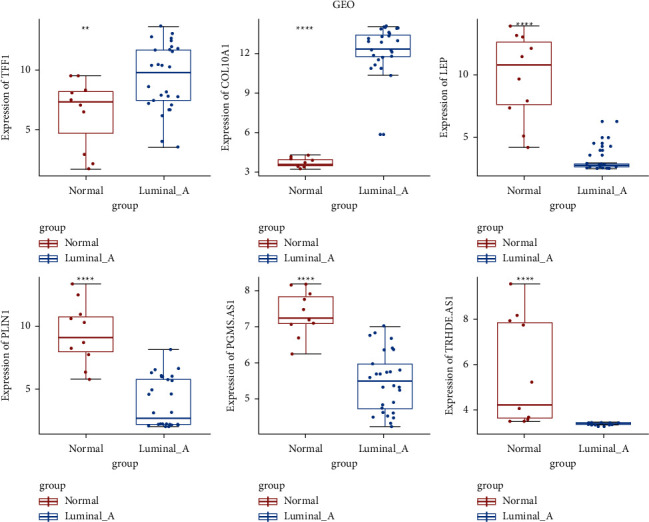
Expression validation of selected DEGs in GSE65194.

**Figure 9:**
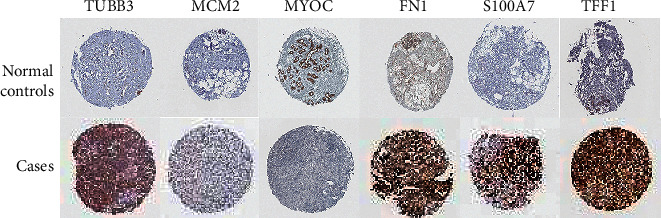
Protein expression validation of selected DEGs in The Human Protein Atlas database.

The mRNA expression patterns of four DEmRNAs (upregulated TFF1 and COL10A1 and downregulated LEP and PLIN1) and two downregulated lncRNAs (PGM5-AS1 and TRHDE-AD1) were verified in the GSE65194 dataset. As shown in Figure 8, mRNA expression of TFF1 and COL10A1 was increased and, LEP, PLIN1, PGM5.A S1, and TRHDE-AD1 were decreased, which was consistent with our RNA sequencing result. In addition, an online immunohistochemical study of selected DEmRNAs (TUBB3, MCM2, MYOC, FN1, S100A7, and TFF1) was conducted using The Human Protein Atlas database (Figure 9). The result showed that the protein expression of TUBB3, MCM2, FN1, S100A7, and TFF1 was increased, and the protein expression of MYOC was downregulated in luminal A breast cancer, which was consistent with the result of RNA expression.

### 3.8. ROC Curve Analyses

**Figure 10:**
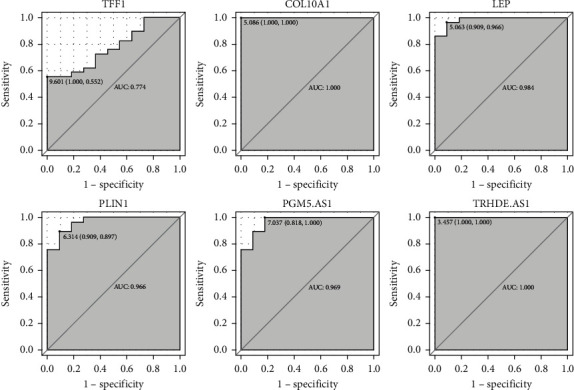
ROC curve. The *x*-axis indicated 1−specificity, and *y*-axis indicated sensitivity.

By using GSE65194, we performed ROC curve analysis to access the diagnostic value of four DEmRNAs (TFF1, COL10A1, LEP, and PLIN1) and two DEmRNAs (PGM5-AS1 and TRHDE-AD1) in luminal A breast cancer. The results indicated that except for TFF1 (AUC = 0.774), COL10A1 (AUC = 1.000), LEP (AUC = 0.984), PLIN1 (AUC = 0.966), PGM5-AS1 (AUC = 0.969), and TRHDE-AD1 (AUC = 1.000) were capable of discriminating luminal A breast cancer and normal controls (Figure 10).

## 4. Discussion

Although some evidence has demonstrated aberrant expression of lncRNA in breast cancer [15], a few studies have systematically examined the function of lncRNA in luminal A breast cancer. In this study, 1451 DElncRNAs and 272 DEmRNAs were identified in luminal A breast cancer. Then, we constructed a luminal A breast cancer-specific PPI, DElncRNA-DEmRNA coexpression, and DElncRNA-nearby DEmRNA interaction networks. In addition, functional analysis of DEmRNAs was performed. We also performed the validation experiment and ROC curve analysis.

COL10A1, a collagen-type *X* alpha 1 chain, is elevated in multiple cancers including the lung, stomach, breast, colon, pancreas, and bladder [16]. Herein, COL10A1 was also upregulated in our RNA sequencing and GSE65194 dataset. In addition, we identified COL10A1 as potential diagnostic biomarkers of luminal A breast cancer. CLEC3A, a C-type lectin domain family 3 member A, has been reported in human breast cancer [17]. It is reported that CLEC3A expression is markedly higher in breast invasive ductal cancer tissues, and elevated CLEC3A expression may be correlated with breast invasive ductal cancer metastatic potential [18]. In this study, CLEC3A, as top 10 DEmRNAs, was also upregulated in our RNA sequencing. Our results are further supported by the fact that CLEC3A is a promising treatment target for breast cancer.

TFF1, trefoil factor 1, is cloned from the breast cancer cell line MCF-7. Smid et al. reported that TFF1 could play an important role in tumor metastasis by binding to cysteine-rich enteroprotein 1 in metastatic breast cancer [19]. Markićević et al. revealed that TFF1 expression levels were significantly higher in breast tissue samples in premenopausal patients [20]. Ishibashi et al. reported that serum TFF1 was significantly higher in women with breast cancer [21]. Elnagdy et al. reported TFF expression levels were markedly higher in blood from breast cancer patients with metastatic disease [22]. In our study, TFF1, as top 10 DEmRNA, was also upregulated in our RNA sequencing results, GSE65194 dataset, and The Human Protein Atlas database. We speculated that serum TFF1 could be a diagnostic biomarker of breast cancer.

DSCAM-AS1, down syndrome cell adhesion molecule antisense lncRNA, is a member of the immunoglobulin superfamily of cell adhesion molecules [23]. Previous studies have indicated that DSCAM-AS1 is involved in the progression of cancer cells [24, 25]. Yashar et al. reported that DSCAM-AS1 could interact with hnRNPL to mediate tumor progression [24]. DSCAM-AS1 enhances non-small cell lung cancer cell migration by elevating BCL11A, and DSCAM-AS1 may be a potential therapeutic target for non-small cell lung cancer [26]. Overexpression of DSCAM-AS1 promotes the cell proliferation of the luminal breast cancer cell line [25]. DSCAM-AS1 is upregulation in invasive ductal carcinoma of the breast and has potential as the diagnostic biomarker [27]. It is suggested that DSCAM-AS1 can be acted as a competing endogenous RNA of miR-137 and regulate EPS8 to accelerate cell reproduction in tamoxifen-resistant breast cancer [28]. Of note, DSCAM-AS1 promotes cell growth and impairs apoptosis of breast cancer cells via reducing miR-204-5p and enhancing RRM2 expression [29]. In the present study, DSCAM-AS1 was top 10 DElncRNA and was upregulated in our RNA sequencing results. However, the mechanism of DSCAM-AS1 in luminal A breast cancer progression largely remains unknown. Further study of DSCAM-AS1-regulated networks is needed.

GPD1, glycerol-3-phosphate dehydrogenase 1, is considered a key element that connects carbohydrate and lipid metabolism. The expression level of GPD1 is significantly downregulated in human breast cancer patients, and overexpression of GPD1 markedly suppresses cell proliferation, migration, and invasion of breast cancer cells [30]. PLIN1, perilipin-1, has been considered as a candidate gene that contributes to the polygenic disease phenotype of human obesity [31]. PLIN1 is markedly declined in human breast cancer, and overexpression of PLIN1 in human breast cancer cells dramatically suppresses cell proliferation, invasion, and *in vivo* tumorigenesis [32]. In our study, PLIN1 was downregulated in our RNA sequencing and GSE65194 dataset and was capable of discriminating luminal A breast cancer and normal controls. Based on the DElncRNA-DEmRNA interaction network, GPD1 and PLIN1 were coexpressed with AC245452.1. Therefore, the study suggested that AC245452.1 may be involved in the occurrence of luminal A breast cancer by regulating the GPD1 and PLIN1.

Interestingly, a total of 4 lncRNA-mRNA pairs were identified between the DElncRNA-nearby DEmRNA interaction network and the DElncRNA-DEmRNA coexpression network, including HOXC-AS3-HOXC10, AC020907.2-FXYD1, AC026461.1-MT1X, and AC132217.1-IGF2. The significantly upregulated expression level of HOXC-AS3 is found in tissues of breast cancer, and the expression of HOXC-AS3 is well associated with the prognosis of breast cancer [33]. AC020907.2 is positively related to overall survival in glioma [34]. Correlation analysis between AC026461.1 and has-miR-330-5p has been found in ovarian cancer [35]. HOXC10 is implicated in luminal breast cancer [36]. It is found that FXYD1 is downregulated in breast tumors [37]. MT1X is upregulated in young breast cancer patients [38]. High IGF2 gene expression in cancer-associated fibroblasts from luminal breast carcinomas is significantly related to a shortened relapse-free survival [39]. This suggested that HOXC10, FXYD1, MT1X, and IGF2 may play an important role in the development of luminal A breast cancer under the regulation of HOXC-AS3, AC020907.2, AC026461.1, and AC132217.1.

In addition, based on functional enrichment of DEmRNAs coexpressed with DElncRNAs, we found that AMPK (involved LIPE and LEP) and PPAR (involved PLIN1) were two significantly enriched pathways in luminal A breast cancer. It is found that targeting AMPK regulatory processes at points can provide additional approaches to prevent breast cancer neoplasia, growth, and metastasis [40]. Apostoli et al. found that the downregulation of PPAR*γ* expression led to a probreast tumorigenic environment [41].

## 5. Conclusions

We identified 1451 DEmRNAs and 272 DElncRNAs in luminal A breast cancer. The expression validation of TFF1, COL10A1, and LEP and PLIN1, PGM5.AS1, and TRHDE-AD1 in the GSE98793 dataset was consistent with the RNA sequencing result. COL10A1, LEP, PLIN1, PGM5-AS1, and TRHDE-AD1 were capable of discriminating luminal A breast cancer and normal controls. In addition, lncRNA-mRNA pairs of HOXC-AS3-HOXC10, AC020907.2-FXYD1, AC026461.1-MT1X, and AC132217.1-IGF2 may be involved in the process of luminal A breast cancer. Our results may be helpful in improving the understanding of the mechanisms associated with the pathogenesis and progression of luminal A breast cancer. However, there are a few limitations in our study. First, the sample size in the RNA sequencing was small and a larger number of luminal A breast cancer samples are further needed. Second, biological functions of identified DEmRNAs and DElncRNAs in luminal A breast cancer were not studied. Therefore, some experiments are needed to investigate the biological function of these molecules in luminal A breast cancer in future work.

## Figures and Tables

**Table 1:** Patient characteristics.

	Case 1	Case 2	Case 3	Case 4
Age (years)	54	64	69	48
Gender	Female	Female	Female	Female
TNM stage	T2N0M0	T1N1M0	T1N0M0	T2N0M0
Luminal	*A*	*A*	*A*	*A*
ER	Positive	Positive	Positive	Positive
PR	Positive	Positive	Positive	Positive
HER-2	Negative	Negative	Negative	Negative
KI-67 (%)	5	10	10	10

ER: Estrogen receptor; PR: progesterone receptor; HER-2: epidermal growth factor receptor; KI-67: rabbit monoclonal antibody against human proliferating nuclear antigen.

**Table 2:** Top 20 DEmRNAs in luminal A breast cancer.

Id	Symbol	Log2FC	*p* value	Up/down
ENSG00000143556	S100A7	−8.61316	7.13*E* − 05	Up
ENSG00000166509	CLEC3A	−8.392	2.19*E* − 06	*p*
ENSG00000260342	AC138811.2	−7.78976	7.21*E* − 06	Up
ENSG00000160182	TFF1	−7.65104	6.97*E* − 08	Up
ENSG00000123500	COL10A1	−7.62325	2.28*E* − 11	Up
ENSG00000185559	DLK1	−7.18485	8.98*E* − 05	Up
ENSG00000170099	SERPINA6	−7.12361	8.66*E* − 06	Up
ENSG00000198077	CYP2A7	−7.03565	1.09*E* − 06	Up
ENSG00000169900	PYDC1	−6.69827	4.83*E* − 06	Up
ENSG00000255974	CYP2A6	−6.25747	0.000592	Up
ENSG00000269711	AC008763.3	7.968183	8.15*E* − 06	Down
ENSG00000174697	LEP	6.90208	1.52*E* − 06	Down
ENSG00000126545	CSN1S1	6.556175	0.002669	Down
ENSG00000167588	GPD1	6.259698	3.25*E* − 07	Down
ENSG00000198759	EGFL6	5.893119	0.001316	Down
ENSG00000186458	DEFB132	5.797332	0.000108	Down
ENSG00000166819	PLIN1	5.791598	2.79*E* − 07	Down
ENSG00000187288	CIDEC	5.788548	4.01*E* − 07	Down
ENSG00000184811	TUSC5	5.739441	9.01*E* − 07	Down
ENSG00000181092	ADIPOQ	5.634258	1.52*E* − 06	Down

FC: fold change.

**Table 3:** Top 20 DElncRNAs in luminal A breast cancer.

Id	Symbol	Log2FC	*p* value	Up/down
ENSG00000235123	DSCAM-AS1	8.203022	0.000251	Up
ENSG00000230838	LINC01614	7.722278	3.24*E* − 06	Up
ENSG00000271850	LINC02343	7.242492	3.54*E* − 06	Up
ENSG00000269353	AC092071.1	6.841357	0.001415	Up
ENSG00000204832	ST8SIA6-AS1	6.801516	0.000343	Up
ENSG00000224271	AL117329.1	6.791899	2.21*E* − 07	Up
ENSG00000260019	LINC01992	6.560384	0.000215	Up
ENSG00000253154	AC100801.1	5.882224	0.003008	Up
ENSG00000262979	AC124319.1	5.882066	0.002757	Up
ENSG00000249346	LINC01016	5.863334	0.000177	Up
ENSG00000267653	AC002546.1	−9.00348	3.38*E* − 05	Down
ENSG00000259760	AC015660.3	−7.46125	0.049795	Down
ENSG00000227591	AL031316.1	−7.24521	0.000474	Down
ENSG00000260877	AP005233.2	−7.1445	0.045135	Down
ENSG00000224958	PGM5-AS1	−7.02679	0.000623	Down
ENSG00000259590	AC015660.2	−6.97901	0.03741	Down
ENSG00000233639	LINC01158	−6.877	0.001136	Down
ENSG00000272254	AC022893.3	−6.80698	0.014414	Down
ENSG00000243961	PARAL1	−6.68908	0.000178	Down
ENSG00000250266	LINC01612	−6.49197	0.021591	Down

FC: fold change.

**Table 4:** DElncRNAs-nearby DEmRNA pairs.

Chr	lncRNA			mRNA		
	Symbol	Start	End	Symbol	Start	End
2	C2orf48	10141382	10211725	AC007240.1	10122739	10199288
11	WT1-AS	32435518	32458769	WT1	32387775	32435630
19	LIPE-AS1	42397128	42652355	LIPE	42401507	42427426
13	LINC00598	40079106	40535807	FOXO1	40469953	40666597
19	AC020907.2	35138824	35151336	FXYD1	35138808	35143109
19	AC020907.2	35138824	35151336	LGI4	35124513	35142451
5	C5orf66	135033280	135358219	PITX1	135027735	135034813
9	PGM5-AS1	68355189	68357852	PGM5	68328308	68531061
1	AL031316.1	209661364	209724125	G0S2	209675420	209676388
1	HSD11B1-AS1	209661364	209724125	HSD11B1	209686178	209734950
1	AL139220.2	44030443	44115913	SLC6A9	43991500	44031467
17	HOXB-AS3	48549630	48606414	HOXB3	48548870	48604912
2	AC104809.2	240954617	240967451	CROCC2	240906330	240993311
12	TRHDE-AS1	72253508	72274907	TRHDE	72087266	72670757
9	VLDLR-AS1	2421597	2643359	VLDLR	2621834	2660053
3	PDZRN3-AS1	73621713	73626796	PDZRN3	73382433	73624940
11	AC132217.1	2129121	2129964	IGF2	2129112	2149603
2	AC108025.2	5696220	5708095	SOX11	5692667	5701385
3	ALDH1L1-AS2	126180065	126210169	ALDH1L1	126103562	126197994
17	AC091180.2	49361165	49369998	ZNF652	49289206	49362473
4	AC097534.1	173322206	173329694	GALNT7	173168753	173323967
12	HOXC-AS3	53981509	53985519	HOXC10	53985065	53990279
7	AC007036.3	30157531	30159534	MTURN	30134810	30162762
12	AC025423.4	68841288	68843237	CPM	68842197	68971570
12	AC079174.1	106245613	106246956	CKAP4	106237877	106304279
X	RHOXF1-AS1	120036236	120146854	RHOXF1	120109053	120115937
15	AC106028.2	92805770	92808567	FAM174B	92617443	92809884
16	AC026461.1	56682470	56687807	MT1X	56682424	56684196
17	AC124319.1	80315651	80316633	RNF213	80260866	80398786
19	AC005546.1	13131571	13132410	NACC1	13118103	13141141
17	RNF157-AS1	76140556	76154650	FOXJ1	76136333	76141299
17	RNF157-AS1	76140556	76154650	RNF157	76142453	76240373
1	LINC01140	87129765	87169198	AC093155.3	86993009	87169204
17	AC002546.1	69477139	69501755	MAP2K6	69414698	69553861
19	AC074135.1	23075201	23100361	ZNF730	23075210	23147219
19	AC011503.1	23919081	23957930	ZNF726	23914876	23945159
15	AC118658.2	98660210	98660668	IGF1R	98648539	98964530

## Data Availability

All data are available in the article.

## Supplementary Materials

Supplementary Materials
*Supplementary Table 1*. Positively coexpressed lncRNA-mRNA network. *Supplementary Table 2*. Negatively coexpressed lncRNA-mRNA network.
