# Supplementary material for: Identification of Crucial lncRNAs for Luminal A Breast Cancer through RNA Sequencing
Source: Int J Endocrinol. 2022 Jun 2;2022:6577942. doi: 10.1155/2022/6577942 (PMC9184229; doi:10.1155/2022/6577942)
Supplement: Supplementary Materials — Supplementary Table 1. Positively coexpressed lncRNA-mRNA network. Supplementary Table 2. Negatively coexpressed lncRNA-mRNA network. [file 6577942.f1.zip › 6577942.f1/Supplementary Table 1.pdf]

mRNAsig	lncRNAsig
RRAGD	LINC01697
LGI4	LINC01697
LIFR	LINC01697
F8	LINC01697
CRYBG3	LINC01697
JAM2	LINC01697
KDR	LINC01697
SHE	LINC01697
TACR1	AC097534.1
NTRK3	AC097534.1
MAB21L1	AC097534.1
TNXB	AC097534.1
SPRY2	AC097534.1
ZDBF2	AC097534.1
CHL1	AC097534.1
PRR26	AC097534.1
ITM2A	AC097534.1
PLEKHM3	AC097534.1
MAP2K6	AC108477.1
LYVE1	LINC00968
CLEC3B	LINC00968
CACNA2D1	LINC00968
BMP2	LINC00968
MAOA	LINC00968
ACSM5	LINC00968
KLHL31	LINC00968
ANGPTL4	LINC00968
AC093155.	LINC00968
ACACB	LINC00968
FAM89A	LINC00968
MTURN	LINC00968
MDFIC	LINC00968
RASL10B	LINC00968
ZFP36	LINC00968
GNG2	LINC00968
ENPP2	LINC00968
NMB	LINC00968
SYNE3	LINC00968
GFRA2	LINC00968
SLC35G2	LINC00968
DTX1	LINC00968
RNF157	LINC00968
SOCS3	LINC00968
DEPP1	LINC00968
CCDC69	LINC00968
PALMD	LINC00968
CD34	LINC00968
TLN2	LINC00968
C19orf12	LINC00968
ACO1	LINC00968

CAT	LINC00968
PALM2-AKA	LINC00968
SPTBN1	LINC00968
ARHGEF6	LINC00968
ITSN1	LINC00968
F8	LINC00968
PPP2R1B	LINC00968
GYPC	LINC00968
ECHDC3	LINC00968
ANTXR2	LINC00968
VEGFB	LINC00968
FERMT2	LINC00968
ADIRF	LINC00968
CAMK1	LINC00968
PLPP3	LINC00968
RAPGEF3	LINC00968
GDPD5	LINC00968
NEK7	LINC00968
TNIP1	LINC00968
CRYBG3	LINC00968
JAM2	LINC00968
TMEM64	LINC00968
DCUN1D3	LINC00968
CDKN1A	LINC00968
ZFAND5	LINC00968
ANXA5	LINC00968
CARD6	LINC00968
MYO1C	LINC00968
UGP2	LINC00968
TNFAIP8	LINC00968
SH3GLB1	LINC00968
PDE11A	LINC00968
DHDDS	LINC00968
ETFDH	LINC00968
KIF1C	LINC00968
C2CD2	LINC00968
SHE	LINC00968
TACC1	LINC00968
CD99L2	LINC00968
PCYOX1	LINC00968
TFF1	AC055854.1
LRRC15	AC055854.1
ASF1B	AC055854.1
DIO2	AC055854.1
CKS2	AC055854.1
HIST3H2BE	AC055854.1
CFL1	AC055854.1
ZNF92	AC055854.1
SP110	AC055854.1
DPCD	AC055854.1
HSD17B13	LINC02202

CALB2	LINC02202
AKR1C1	LINC02202
LYVE1	LINC02202
ITIH5	LINC02202
APOB	LINC02202
RDH5	LINC02202
ADRA1A	LINC02202
NPY5R	LINC02202
AKR1C3	LINC02202
CLEC3B	LINC02202
ANGPT1	LINC02202
CACNA2D1	LINC02202
CAV2	LINC02202
SORBS1	LINC02202
SEMA3G	LINC02202
BMP2	LINC02202
MT1M	LINC02202
ITGA7	LINC02202
ACACB	LINC02202
EBF1	LINC02202
MDFIC	LINC02202
TNS1	LINC02202
CAV1	LINC02202
DDR2	LINC02202
TMEM100	LINC02202
MCAM	LINC02202
CLMP	LINC02202
SLC35G2	LINC02202
C8orf34	LINC02202
DTX1	LINC02202
RNF157	LINC02202
GSN	LINC02202
PALM	LINC02202
DEPP1	LINC02202
PALMD	LINC02202
SIK2	LINC02202
CAT	LINC02202
DSEL	LINC02202
SEMA3A	LINC02202
SYNPO	LINC02202
ARHGEF6	LINC02202
RGCC	LINC02202
EPDR1	LINC02202
PPP2R1B	LINC02202
EPAS1	LINC02202
NMT2	LINC02202
ACSS2	LINC02202
TGFBR2	LINC02202
FERMT2	LINC02202
PLPP3	LINC02202
ADRB2	LINC02202

TEAD1	LINC02202
CCND2	LINC02202
STAT5A	LINC02202
CDKN1A	LINC02202
CARD6	LINC02202
BIN1	LINC02202
SH3D19	LINC02202
CRTAP	LINC02202
ARHGAP42	LINC02202
C2CD2	LINC02202
TACC1	LINC02202
FOXO1	LINC02202
ITPRIP	LINC02202
CD300LG	AC110597.1
AQP7	AC110597.1
MYZAP	AC110597.1
PQLC2L	AC110597.1
MLXIPL	AC110597.1
CXCL2	AC110597.1
FIGN	AC110597.1
SLC10A6	AC110597.1
WDR49	AC110597.1
KCNJ8	AC110597.1
MME	AC110597.1
CYYR1	AC110597.1
CLIC5	AC110597.1
CHST3	AC110597.1
FGD4	AC110597.1
FAM171A1	AC110597.1
MAMDC2	AC110597.1
PGRMC2	AC110597.1
PDGFD	AC110597.1
KAT2B	AC110597.1
ALAD	AC110597.1
PLSCR4	AC110597.1
UST	AC110597.1
KDR	AC110597.1
SAT2	AC110597.1
EZH1	AC110597.1
ZDHHC2	AC110597.1
PCOLCE2	RHOXF1-AS1
G0S2	RHOXF1-AS1
LIPE	RHOXF1-AS1
CIDEA	RHOXF1-AS1
C14orf18C	RHOXF1-AS1
LGALS12	RHOXF1-AS1
PFKFB1	RHOXF1-AS1
SLC7A10	RHOXF1-AS1
AGPAT2	RHOXF1-AS1
PC	RHOXF1-AS1
BTG2	RHOXF1-AS1

ACKR3	RHOXF1-AS1
GPD1	TRHDE-AS1
PLIN1	TRHDE-AS1
CIDEC	TRHDE-AS1
TUSC5	TRHDE-AS1
ADIPOQ	TRHDE-AS1
LPL	TRHDE-AS1
PLIN4	TRHDE-AS1
FABP4	TRHDE-AS1
RBP4	TRHDE-AS1
HSPB7	TRHDE-AS1
G0S2	TRHDE-AS1
LIPE	TRHDE-AS1
SAA1	TRHDE-AS1
NAT8L	TRHDE-AS1
HEPACAM	TRHDE-AS1
CALB2	TRHDE-AS1
ALDH1L1	TRHDE-AS1
AOC3	TRHDE-AS1
SLC19A3	TRHDE-AS1
ITIH5	TRHDE-AS1
APOB	TRHDE-AS1
SLC16A7	TRHDE-AS1
RDH5	TRHDE-AS1
S100B	TRHDE-AS1
KCNIP2	TRHDE-AS1
SLC2A4	TRHDE-AS1
ANGPT1	TRHDE-AS1
CAV2	TRHDE-AS1
SORBS1	TRHDE-AS1
NPR1	TRHDE-AS1
FHL1	TRHDE-AS1
KLF15	TRHDE-AS1
ITGA7	TRHDE-AS1
GHR	TRHDE-AS1
TNS1	TRHDE-AS1
DUSP1	TRHDE-AS1
NPR3	TRHDE-AS1
FOS	TRHDE-AS1
MCAM	TRHDE-AS1
CLMP	TRHDE-AS1
GPT	TRHDE-AS1
RARRES2	TRHDE-AS1
HOXA10	TRHDE-AS1
SIK2	TRHDE-AS1
SYNPO	TRHDE-AS1
ARHGEF6	TRHDE-AS1
NIPSNAP3E	TRHDE-AS1
ACSS2	TRHDE-AS1
DHRS3	TRHDE-AS1
ANGPTL2	TRHDE-AS1

NATD1	TRHDE-AS1
PGRMC2	TRHDE-AS1
GABARAPL1	TRHDE-AS1
TEAD1	TRHDE-AS1
KAT2B	TRHDE-AS1
ALAD	TRHDE-AS1
FAM83D	AP000439.2
CEP55	AP000439.2
TOP2A	AP000439.2
KIF14	AP000439.2
HIST1H2AL	AP000439.2
FAM111B	AP000439.2
CDCA5	AP000439.2
RAD54L	AP000439.2
CDCA2	AP000439.2
UHRF1	AP000439.2
NDC80	AP000439.2
HPX	AP000439.2
HIST1H2AM	AP000439.2
NUF2	AP000439.2
KIFC1	AP000439.2
KNL1	AP000439.2
CDCA8	AP000439.2
GTSE1	AP000439.2
FRMPD3	AP000439.2
KIF20A	AP000439.2
FAM72D	AP000439.2
AURKA	AP000439.2
FANCI	AP000439.2
ECT2	AP000439.2
RECQL4	AP000439.2
HIST1H2BH	AP000439.2
OTUB2	AP000439.2
CCNB1	AP000439.2
MCM4	AP000439.2
HIST2H2AA	AP000439.2
HIST2H2AA	AP000439.2
MCM2	AP000439.2
PAQR4	AP000439.2
ENTPD7	AP000439.2
SMC4	AP000439.2
PDIA4	AP000439.2
SLC12A8	AP000439.2
SEPHS2	AP000439.2
SLC20A1	AP000439.2
DGKD	AP000439.2
YIPF1	AP000439.2
NCAPG2	AP000439.2
FANCD2	AP000439.2
GJB2	AL139220.2
WISP1	AL139220.2

NAT1	AL139220.2
RAMP1	AL139220.2
CLEC5A	AL139220.2
HPN	AL139220.2
B4GALNT4	AL139220.2
TIMP1	AL139220.2
MCRIP2	AL139220.2
CYP4Z1	LINC01906
F2RL2	LINC01906
NAT1	LINC01906
HPN	LINC01906
OAS2	LINC01906
OASL	LINC01906
MUC1	LINC01906
E2F7	AL133373.2
ZNF138	AL133373.2
HOXC13	HOXC-AS3
HOXC10	HOXC-AS3
UTP20	HOXC-AS3
CIDEC	AL078639.1
TUSC5	AL078639.1
ADIPOQ	AL078639.1
FABP4	AL078639.1
RBP4	AL078639.1
NNAT	AL078639.1
AOC3	AL078639.1
SLC19A3	AL078639.1
SLC16A7	AL078639.1
KCNIP2	AL078639.1
RARRES2	AL078639.1
SIX2	LINC00473
FAM72B	LINC00473
CPB1	AP005121.1
GRIA2	AP005121.1
FOXJ1	AP005121.1
MATN3	AP005121.1
ADAMTS16	AP005121.1
TPH1	AP005121.1
SFRP2	AP005121.1
EVL	AP005121.1
OAS3	AP005121.1
LOXL1	AP005121.1
OASL	AP005121.1
PYCARD	AP005121.1
IRF7	AP005121.1
OAS1	AP005121.1
SLC40A1	AP005121.1
FGD3	AP005121.1
REPS2	AP005121.1
SAMD9	AP005121.1
PARP9	AP005121.1

NUP210	AP005121.1
CCR5	AC005837.1
AC093155.	LINC01230
SGK2	LINC01230
RASL10B	LINC01230
NPR3	LINC01230
RNF157	LINC01230
CDKN2C	LINC01230
PKDCC	LINC01230
EPAS1	LINC01230
VKORC1L1	LINC01230
GDPD5	LINC01230
TEAD1	LINC01230
CDKN1A	LINC01230
CBX2	MEG8
CDKN3	AL356740.2
CCR5	AL356740.2
F2RL2	AL139412.1
RASL11B	AL139412.1
MUC1	AL139412.1
TPRN	AL139412.1
UBXN10	AL139412.1
RAI14	AL139412.1
RALGPS2	AL139412.1
FAM174B	AC091544.4
TTC39A	AC091544.4
ORC1	AL645608.1
PRR11	AL645608.1
SDR16C5	AL645608.1
ADAMTS17	AL645608.1
GALNT7	AL645608.1
UCP2	AL645608.1
TMEM241	AL645608.1
CTPS1	AL645608.1
ADSS	AL645608.1
KIF14	LINC00664
HSPB7	AP001528.2
SAA1	AP001528.2
CALB2	AP001528.2
LYVE1	AP001528.2
AOC3	AP001528.2
CHRDL1	AP001528.2
RDH5	AP001528.2
AKR1C3	AP001528.2
CLEC3B	AP001528.2
ANGPT1	AP001528.2
CACNA2D1	AP001528.2
SEMA3G	AP001528.2
NPR1	AP001528.2
GPX3	AP001528.2
ACSM5	AP001528.2

ITGA7	AP001528.2
FAM89A	AP001528.2
EBF1	AP001528.2
MTURN	AP001528.2
MDFIC	AP001528.2
TNS1	AP001528.2
CAV1	AP001528.2
DDR2	AP001528.2
AKAP12	AP001528.2
ANGPTL1	AP001528.2
TMEM100	AP001528.2
EBF3	AP001528.2
GNG2	AP001528.2
MCAM	AP001528.2
NLRP3	AP001528.2
SYNE3	AP001528.2
GFRA2	AP001528.2
COX7A1	AP001528.2
DTX1	AP001528.2
PLA2G16	AP001528.2
AOX1	AP001528.2
GSN	AP001528.2
PALM	AP001528.2
RARRES2	AP001528.2
DEPP1	AP001528.2
CCDC69	AP001528.2
SVEP1	AP001528.2
TLCD2	AP001528.2
PALMD	AP001528.2
CPED1	AP001528.2
TWIST2	AP001528.2
C19orf12	AP001528.2
KLF4	AP001528.2
CAT	AP001528.2
PALM2-AKA	AP001528.2
AFAP1L1	AP001528.2
SYNPO	AP001528.2
ARHGEF6	AP001528.2
RGCC	AP001528.2
LAMA4	AP001528.2
RILP	AP001528.2
VIM	AP001528.2
MAP7D3	AP001528.2
ECHDC3	AP001528.2
DLC1	AP001528.2
TGFBR2	AP001528.2
DHRS3	AP001528.2
FERMT2	AP001528.2
CAMK1	AP001528.2
PNPLA2	AP001528.2
SNTA1	AP001528.2

CCND2	AP001528.2
TIMP3	AP001528.2
NEK7	AP001528.2
CSPG4	AP001528.2
CDKN1A	AP001528.2
ANXA5	AP001528.2
MYO1C	AP001528.2
ACAT1	AP001528.2
SH3D19	AP001528.2
TK2	AP001528.2
CRTAP	AP001528.2
ARHGAP42	AP001528.2
TACC1	AP001528.2
ITPRIP	AP001528.2
PPP2R2C	LINC02303
SGO1	LINC02303
MAK	LINC02303
ADAMDEC1	LINC02303
AC007325.	LINC02303
CCNE2	LINC02303
E2F7	LINC02303
CKAP2	LINC02303
ZNHIT2	LINC02303
MICAL2	LINC02303
CFL1	LINC02303
ZNF253	LINC02303
ADTRP	AL117190.1
COG6	AL117190.1
HIST1H2AI	AL354707.1
MYBL2	AL354707.1
ANLN	AL354707.1
CXCL9	AL354707.1
CENPF	AL354707.1
CDC45	AL354707.1
ORC1	AL354707.1
BUB1	AL354707.1
HIST1H2BL	AL354707.1
HMGB3	AL354707.1
FAM72D	AL354707.1
PRR11	AL354707.1
KIF23	AL354707.1
HIST1H2BE	AL354707.1
E2F1	AL354707.1
BRIP1	AL354707.1
SLC6A9	AL354707.1
HIST1H3D	AL354707.1
DNAH14	AL354707.1
SERPINE2	AL354707.1
ATP6V0B	AL354707.1
TMEM206	AL354707.1
HIST1H4K	AL354707.1

HIST1H4J	AL354707.1
CIP2A	AL354707.1
RAB3D	AL354707.1
MAGED1	AL354707.1
SRD5A3	AL354707.1
SHMT2	AL354707.1
SLC37A1	AL354707.1
S100A11	AL354707.1
NCAPG2	AL354707.1
RNF182	MEG9
C4A	LINC00536
EVL	MRPS30-DT
LONRF2	MRPS30-DT
TRAF5	MRPS30-DT
CNTFR	AC020907.2
SCARA5	AC020907.2
FXYD1	AC020907.2
FMO2	AC020907.2
F10	AC020907.2
TEF	AC020907.2
MYC	AC020907.2
FAM83D	AC074135.1
NUSAP1	AC074135.1
FRMPD3	AC074135.1
SLC30A8	AC074135.1
DIAPH3	AC074135.1
CIT	AC074135.1
E2F2	AC074135.1
CENPK	AC074135.1
TMC5	AC074135.1
CHEK1	AC074135.1
KIAA1211	AC068669.1
DPT	LINC01140
AKR1C3	LINC01140
CLEC3B	LINC01140
ANGPT1	LINC01140
CACNA2D1	LINC01140
SEMA3G	LINC01140
ITGA7	LINC01140
MDFIC	LINC01140
CAV1	LINC01140
DDR2	LINC01140
GALNT13	LINC01140
EBF3	LINC01140
GNG2	LINC01140
NLRP3	LINC01140
SYNE3	LINC01140
GFRA2	LINC01140
COX7A1	LINC01140
PLA2G16	LINC01140
GSN	LINC01140

PALM	LINC01140
DEPP1	LINC01140
CCDC69	LINC01140
TLCD2	LINC01140
GPR146	LINC01140
C19orf12	LINC01140
CAT	LINC01140
PALM2-AKA	LINC01140
AFAP1L1	LINC01140
LAMA4	LINC01140
RILP	LINC01140
AIFM2	LINC01140
VEGFB	LINC01140
SMIM3	LINC01140
FERMT2	LINC01140
CAMK1	LINC01140
PNPLA2	LINC01140
SNTA1	LINC01140
RASA3	LINC01140
CCND2	LINC01140
TIMP3	LINC01140
NEK7	LINC01140
TNIP1	LINC01140
CSPG4	LINC01140
ANXA5	LINC01140
PLOD2	LINC01140
MYO1C	LINC01140
ACAT1	LINC01140
SH3D19	LINC01140
TK2	LINC01140
CRTAP	LINC01140
EPHX1	LINC01140
C2CD2	LINC01140
CD99L2	LINC01140
ITPRIP	LINC01140
SAA1	AP001528.3
CFD	AP001528.3
CD300LG	AP001528.3
ALDH1L1	AP001528.3
ITIH5	AP001528.3
APOB	AP001528.3
CNTFR	AP001528.3
BTNL9	AP001528.3
CAV2	AP001528.3
SORBS1	AP001528.3
BMP2	AP001528.3
SAA2	AP001528.3
ACACB	AP001528.3
EBF1	AP001528.3
DUSP1	AP001528.3
FOS	AP001528.3

SLC35G2	AP001528.3
C8orf34	AP001528.3
DTX1	AP001528.3
GSN	AP001528.3
DEPP1	AP001528.3
PALMD	AP001528.3
CKMT2	AP001528.3
COL25A1	AP001528.3
RGCC	AP001528.3
CLIC5	AP001528.3
NMT2	AP001528.3
TGFBR2	AP001528.3
TEF	AP001528.3
FGD4	AP001528.3
PLPP3	AP001528.3
STAT5A	AP001528.3
KAT2B	AP001528.3
CRYBG3	AP001528.3
ALAD	AP001528.3
JAM2	AP001528.3
UST	AP001528.3
CARD6	AP001528.3
TNFAIP8	AP001528.3
ARHGAP42	AP001528.3
SOD2	AP001528.3
FOXO1	AP001528.3
MMD	LINC01239
PRKAR2B	LINC01239
ACSL1	LINC01239
TRHDE	LINC01239
IRX6	LINC01239
GNAI1	LINC01239
MGLL	LINC01239
NMB	LINC01239
RETSAT	LINC01239
ACO1	LINC01239
SPTBN1	LINC01239
FAM213A	LINC01239
COQ8A	LINC01239
RHOBTB3	LINC01239
GBE1	LINC01239
GRK3	LINC01239
DIAPH2	LINC01239
PTPRS	LINC01239
ITGB1BP1	LINC01239
UGP2	LINC01239
PGM1	LINC01239
PDE11A	LINC01239
PCYOX1	LINC01239
FAM174B	LINC00930
FBXO21	LINC00930

ARL1	LINC00930
NNAT	AC134043.2
SAA1	AC134043.2
CALB2	AC134043.2
ITIH5	AC134043.2
RDH5	AC134043.2
SLC2A4	AC134043.2
CACNA2D1	AC134043.2
SORBS1	AC134043.2
SAA2	AC134043.2
KLF15	AC134043.2
TNS1	AC134043.2
CAV1	AC134043.2
DDR2	AC134043.2
TMEM100	AC134043.2
MCAM	AC134043.2
COX7A1	AC134043.2
AOX1	AC134043.2
GSN	AC134043.2
DEPP1	AC134043.2
SCN9A	AC134043.2
TWIST2	AC134043.2
SIK2	AC134043.2
KLF4	AC134043.2
FAM107A	AC134043.2
CAT	AC134043.2
DSEL	AC134043.2
ARHGEF6	AC134043.2
RGCC	AC134043.2
PPP2R1B	AC134043.2
RILP	AC134043.2
ECHDC3	AC134043.2
NMT2	AC134043.2
TGFBR2	AC134043.2
DHRS3	AC134043.2
FERMT2	AC134043.2
ANGPTL2	AC134043.2
PNPLA2	AC134043.2
GABARAPL1	AC134043.2
FADS3	AC134043.2
STAT5A	AC134043.2
CDKN1A	AC134043.2
ACAT1	AC134043.2
SH3D19	AC134043.2
PLPP1	AC134043.2
TK2	AC134043.2
CRTAP	AC134043.2
ARHGAP42	AC134043.2
TACC1	AC134043.2
FOXO1	AC134043.2
DPT	AC093278.2

SLC35G2	AC093278.2
ADGRF5	AC093278.2
EDNRB	AC093278.2
SLC10A6	AC093278.2
TLN2	AC093278.2
TXNIP	AC093278.2
CDH5	AC093278.2
PLPP3	AC093278.2
SLC30A8	LINC00511
TDG	LINC00511
STK36	LINC00511
G0S2	AC024909.2
NNAT	AC024909.2
S100B	AC024909.2
HADH	AC024909.2
SPTSSB	AC118658.2
IGF1R	AC118658.2
ORC1	AC144450.1
HPX	AC144450.1
GJB1	AC144450.1
ERBB2	AC144450.1
SAPCD2	AC144450.1
RTKN2	AC144450.1
GALNT7	AC144450.1
DHRS13	AC144450.1
MBOAT2	AC144450.1
HID1	AC144450.1
EGLN3	LINC02085
DNAJA4	LINC02085
KLF9	LINC00598
TNMD	PCAT19
ADH1C	PCAT19
LRRC59	AC079310.1
DDX52	AC079310.1
PSMD3	AC079310.1
MSL1	AC079310.1
GJB1	AC079310.1
SLC35B1	AC079310.1
MED24	AC079310.1
XYLT2	AC079310.1
CAPS	AC079310.1
NDC1	AC079310.1
NBEA	AC079310.1
STARD3	AC079310.1
MSH6	AC079310.1
HSPH1	AC079310.1
UTP18	AC079310.1
HMGCS1	AC079310.1
LMNB2	AC079310.1
BMF	AC079310.1
CPSF3	AC079310.1

PGAP3	AC079310.1
DNAJA4	AC079310.1
UBE2T	HOTAIR
LRRC31	HOTAIR
AMMECR1	HOTAIR
CDC7	HOTAIR
OPN3	HOTAIR
HSD17B7	HOTAIR
TRERF1	HOTAIR
ABCA12	AC159540.2
DEPDC1	AC159540.2
RRM2	AC159540.2
SAMD11	AC159540.2
FBN2	AC159540.2
MN1	AC159540.2
ANO7	AC159540.2
TMEM206	AC159540.2
CCDC150	AC159540.2
RAB3D	AC159540.2
MAGED1	AC159540.2
DHRS13	AC159540.2
SRD5A3	AC159540.2
NEMP1	AC159540.2
TTYH3	AC159540.2
HID1	AC159540.2
LAMP5	AC013652.1
ARNT2	AC013652.1
TESMIN	AC013652.1
SYT7	AC013652.1
HSD17B7	AC013652.1
WDR90	AC013652.1
NUP210	AC013652.1
RASL11B	AC016705.2
BPIFB1	AL365181.3
PRCD	AC011379.1
WNT11	AC011379.1
WDR49	AC011379.1
FGD4	AC011379.1
NEK2	RHPN1-AS1
FAM83D	RHPN1-AS1
CEP55	RHPN1-AS1
PKMYT1	RHPN1-AS1
NDC80	RHPN1-AS1
NUF2	RHPN1-AS1
SGO1	RHPN1-AS1
MAK	RHPN1-AS1
CCNE2	RHPN1-AS1
CKAP2	RHPN1-AS1
SLC20A1	RHPN1-AS1
ZNF253	RHPN1-AS1
MYCBP	RHPN1-AS1

CBX2	MEG3
STARD3	MEG3
ITIH5	AC026461.1
PQLC2L	AC026461.1
CAVIN2	AC026461.1
SAA2	AC026461.1
KLF15	AC026461.1
SCARA5	AC026461.1
MT1M	AC026461.1
GHR	AC026461.1
TNS1	AC026461.1
TMEM100	AC026461.1
MYOM1	AC026461.1
FIGN	AC026461.1
MT1X	AC026461.1
P2RY12	AC026461.1
SCN9A	AC026461.1
CRYAB	AC026461.1
LRRN4CL	AC026461.1
NLGN1	AC026461.1
CKMT2	AC026461.1
FAM107A	AC026461.1
DSEL	AC026461.1
RGCC	AC026461.1
CHST3	AC026461.1
TENM1	AC026461.1
CAVIN1	AC026461.1
MMRN2	AC026461.1
TMTC1	AC026461.1
ANXA1	AC026461.1
DLC1	AC026461.1
NMT2	AC026461.1
TGFBR2	AC026461.1
EHD2	AC026461.1
EFEMP1	AC026461.1
TEF	AC026461.1
ADAMTS5	AC026461.1
MPP6	AC026461.1
SOX5	AC026461.1
GNAL	AC026461.1
PROS1	AC026461.1
STAT5A	AC026461.1
IRS2	AC026461.1
KAT2B	AC026461.1
LHFPL6	AC026461.1
ADAMTS1	AC026461.1
CDC42BPA	AC026461.1
SH3D19	AC026461.1
PLPP1	AC026461.1
EZH1	AC026461.1
ZDHHC2	AC026461.1

FOXO1	AC026461.1
PTPRG	AC026461.1
ITPRIP	AC026461.1
PROX1	AC105046.1
ADRB1	FGF14-AS2
CAB39	FGF14-AS2
FFAR2	AC141930.1
SIX2	AC141930.1
KIF1A	AC141930.1
GJB1	AC141930.1
CENPU	AC141930.1
FAM72B	AC141930.1
GALNT7	AC141930.1
NPY5R	AC007036.3
PRKAR2B	AC007036.3
MGLL	AC007036.3
WASF3	AC007036.3
COQ8A	AC007036.3
GRK3	AC007036.3
PTPRS	AC007036.3
ITGB1BP1	AC007036.3
UGP2	AC007036.3
PGM1	AC007036.3
FABP4	AC104986.2
ADH1B	AC104986.2
TMEM132C	AC104986.2
ATP1A2	AC104986.2
SOD3	AC104986.2
SLC22A3	AC104986.2
ECSCR	AC104986.2
PLEKHA8	DGUOK-AS1
ADRA1A	LIPE-AS1
BIN1	LIPE-AS1
TEK	EMX2OS
DAAM2	EMX2OS
PTPRB	EMX2OS
KIF14	AC106820.2
PGM5	AC132217.1
IGFBP6	AC132217.1
IGF2	AC132217.1
SATB1	AC132217.1
CD300LG	AP001816.1
DENND2A	AP001816.1
KCNJ8	AP001816.1
STXBP1	AP001816.1
ALDOC	LINC01550
ADRB1	LINC01550
GPC3	LINC01550
TF	LINC01550
LARP6	LINC01550
RASGEF1B	LINC01550

FBXO8	LINC01550
USP53	LINC01550
CAB39	LINC01550
S100A7	AL645608.3
S100A8	AL645608.3
ERBB2	AL645608.3
SLC35B1	AL645608.3
MRPL27	AL645608.3
XYLT2	AL645608.3
DPH2	AL645608.3
MSH6	AL645608.3
AC023055.	AC009119.1
PRR11	AC009119.1
EZH2	HELLPAR
NDC1	HELLPAR
MYO10	HELLPAR
KDM5B	HELLPAR
FLVCR1	HELLPAR
NEMP1	HELLPAR
TFF3	TERC
PRAME	TERC
HIST1H3I	TERC
KIF24	TERC
LEP	AC020915.3
ADIPOQ	AC020915.3
LPL	AC020915.3
GLYAT	AC020915.3
CD36	AC020915.3
HSD17B13	AC020915.3
AKR1C1	AC020915.3
AOC3	AC020915.3
AKR1C3	AC020915.3
GALNT13	AC020915.3
RBP7	AC020915.3
ECM2	AC020915.3
NACC2	AC020915.3
PDE5A	AL731571.1
CLEC4F	MANEA-AS1
LEP	AC245452.1
GPD1	AC245452.1
PLIN1	AC245452.1
CIDEC	AC245452.1
ADIPOQ	AC245452.1
TIMP4	AC245452.1
LPL	AC245452.1
PLIN4	AC245452.1
FABP4	AC245452.1
GLYAT	AC245452.1
CD36	AC245452.1
HSPB7	AC245452.1
ADH1B	AC245452.1

HSD17B13	AC245452.1
DPT	AC245452.1
TMEM132C	AC245452.1
ALDH1L1	AC245452.1
AOC3	AC245452.1
PCDH9	AC245452.1
APOB	AC245452.1
AKR1C3	AC245452.1
CLEC3B	AC245452.1
ANGPT1	AC245452.1
SEMA3G	AC245452.1
NPR1	AC245452.1
GPX3	AC245452.1
ACSM5	AC245452.1
ITGA7	AC245452.1
EBF1	AC245452.1
CAV1	AC245452.1
MMP28	AC245452.1
EBF3	AC245452.1
SYNE3	AC245452.1
RBP7	AC245452.1
ABCC9	AC245452.1
DTX1	AC245452.1
GSN	AC245452.1
PALM	AC245452.1
RARRES2	AC245452.1
CCDC69	AC245452.1
TLN2	AC245452.1
TXNIP	AC245452.1
LAMA4	AC245452.1
JDP2	AC245452.1
CSPG4	AC245452.1
ACAT1	AC245452.1
NACC2	AC245452.1
CFD	AC048382.5
CD300LG	AC048382.5
ALDH1L1	AC048382.5
ITIH5	AC048382.5
RDH5	AC048382.5
ADRA1A	AC048382.5
BTNL9	AC048382.5
CAVIN2	AC048382.5
KLF15	AC048382.5
SCARA5	AC048382.5
MT1M	AC048382.5
ASPA	AC048382.5
GHR	AC048382.5
PLAC9	AC048382.5
TMEM100	AC048382.5
MYOM1	AC048382.5
SRPX	AC048382.5

FIGN	AC048382.5
MT1X	AC048382.5
P2RY12	AC048382.5
SCN9A	AC048382.5
CRYAB	AC048382.5
LRRN4CL	AC048382.5
ABCA9	AC048382.5
FAM107A	AC048382.5
FREM1	AC048382.5
DSEL	AC048382.5
RGCC	AC048382.5
KANK3	AC048382.5
TENM1	AC048382.5
CAVIN1	AC048382.5
MMRN2	AC048382.5
TMTC1	AC048382.5
DLC1	AC048382.5
NMT2	AC048382.5
TGFBR2	AC048382.5
EHD2	AC048382.5
EFEMP1	AC048382.5
TEF	AC048382.5
FAM171A1	AC048382.5
SOX5	AC048382.5
GNAL	AC048382.5
PROS1	AC048382.5
IRS2	AC048382.5
KAT2B	AC048382.5
SH3D19	AC048382.5
PLPP1	AC048382.5
PTPRG	AC048382.5
ITPRIP	AC048382.5
TUSC5	TRIM52-AS1
RBP4	TRIM52-AS1
NNAT	TRIM52-AS1
HEPACAM	TRIM52-AS1
FHL1	TRIM52-AS1
GHR	TRIM52-AS1
HSPB6	TRIM52-AS1
CLMP	TRIM52-AS1
GPT	TRIM52-AS1
HOXA10	TRIM52-AS1
DHRS3	TRIM52-AS1
ANGPTL2	TRIM52-AS1
MBNL3	TRIM52-AS1
DEPDC1	AC023509.2
CXCL11	AC114271.1
PROX1	AC011247.1
ATP1A2	AC020916.1
FOSB	AC020916.1
PTGS2	AC020916.1

EVL	SEPT4-AS1
AC091057.	LINC01814
KIF18A	LINC01814
PROX1	ZNF667-AS1
MYZAP	AL662844.4
SAA2	AL662844.4
FOS	AL662844.4
EGR1	AL662844.4
C8orf34	AL662844.4
FOSB	AL662844.4
RNF150	AL662844.4
RSPO3	AL662844.4
CKMT2	AL662844.4
MME	AL662844.4
NIPSNAP3E	AL662844.4
ANXA1	AL662844.4
MRAS	AL662844.4
PPP1R15A	AL662844.4
JUN	AL662844.4
KANK1	AL662844.4
CLIP4	AL662844.4
CSRNP1	AL662844.4
ATF3	AL662844.4
ECHDC1	AL662844.4
EZH1	AL662844.4
USP53	AL662844.4
ZDHHC2	AL662844.4
SCARA5	MIR222HG
MT1X	MIR222HG
RSPO3	MIR222HG
APCDD1	MIR222HG
MME	MIR222HG
CHST3	MIR222HG
SPRY2	MIR222HG
ALDH1A2	MIR222HG
ADAMTS5	MIR222HG
MPP6	MIR222HG
ABCA5	MIR222HG
ADGRL2	MIR222HG
CSRP2	MIR222HG
EZH1	MIR222HG
SHANK3	MIR222HG
PQLC2L	RAB11B-AS1
KCNB1	RAB11B-AS1
ALDOC	RAB11B-AS1
ADRB1	RAB11B-AS1
GPC3	RAB11B-AS1
TF	RAB11B-AS1
LARP6	RAB11B-AS1
RNF150	RAB11B-AS1
FAM13A	RAB11B-AS1

KANK1	RAB11B-AS1
CLIP4	RAB11B-AS1
JADE1	RAB11B-AS1
MOCS1	RAB11B-AS1
ECHDC1	RAB11B-AS1
RASGEF1B	RAB11B-AS1
FAM49A	RAB11B-AS1
FBXO8	RAB11B-AS1
USP53	RAB11B-AS1
CAB39	RAB11B-AS1
