# Supplementary material for: Identification of Crucial lncRNAs for Luminal A Breast Cancer through RNA Sequencing
Source: Int J Endocrinol. 2022 Jun 2;2022:6577942. doi: 10.1155/2022/6577942 (PMC9184229; doi:10.1155/2022/6577942)
Supplement: Supplementary Materials — Supplementary Table 1. Positively coexpressed lncRNA-mRNA network. Supplementary Table 2. Negatively coexpressed lncRNA-mRNA network. [file 6577942.f1.zip › 6577942.f1/Supplementary Table 2.pdf]

mRNAsig	lncRNAsig
WDR62	LINC01697
POC1A	LINC01697
AC091057.	LINC01697
TINCR	LINC01697
KIF18A	LINC01697
EGLN3	LINC01697
C19orf48	LINC01697
CORO2A	LINC01697
EXO1	AC097534.1
AGT	AC097534.1
GJB2	AC097534.1
CENPF	AC097534.1
FAM111B	AC097534.1
CDCA5	AC097534.1
UHRF1	AC097534.1
CFB	AC097534.1
UBE2T	AC097534.1
CDC6	AC097534.1
NAT1	AC097534.1
TLCD1	AC097534.1
CDCA8	AC097534.1
DIO2	AC097534.1
BGN	AC097534.1
CKS2	AC097534.1
HIST3H2BE	AC097534.1
ECT2	AC097534.1
DNA2	AC097534.1
ADGRB2	AC097534.1
SLC1A1	AC097534.1
CREB3L1	AC097534.1
SLC39A6	AC097534.1
CDC7	AC097534.1
SULF1	AC097534.1
IRF7	AC097534.1
ZNHIT2	AC097534.1
HIST2H2AA	AC097534.1
TIMP1	AC097534.1
SMC4	AC097534.1
DDX58	AC097534.1
SEPHS2	AC097534.1
CCNF	AC097534.1
F7	AC108477.1
CATSPERB	LINC00968
PAX9	LINC00968
MFSD2A	LINC00968
PPFIA4	LINC00968
POC1A	LINC00968
TMC5	LINC00968
CHEK1	LINC00968
PAFAH1B3	LINC00968

C19orf48	LINC00968
HIST1H1D	LINC00968
PASK	LINC00968
RGL3	LINC00968
SDC1	LINC00968
MDK	LINC00968
HELLS	LINC00968
SLC37A1	LINC00968
CORO2A	LINC00968
FANCD2	LINC00968
MOCS1	AC055854.1
FAM49A	AC055854.1
HPX	LINC02202
PAX9	LINC02202
MFSD2A	LINC02202
PPFIA4	LINC02202
DNAH14	LINC02202
HIST1H2BH	LINC02202
SERPINE2	LINC02202
GINS2	LINC02202
PAFAH1B3	LINC02202
PAQR4	LINC02202
HIST1H1D	LINC02202
TMEM97	LINC02202
HIST1H2BG	LINC02202
PASK	LINC02202
RGL3	LINC02202
MDK	LINC02202
SLC12A8	LINC02202
HELLS	LINC02202
SLC37A1	LINC02202
CORO2A	LINC02202
TFF3	AC110597.1
HIST1H2AI	AC110597.1
HIST1H2AM	AC110597.1
HIST1H3I	AC110597.1
HIST1H2BE	AC110597.1
HIST1H3D	AC110597.1
RFC5	AC110597.1
ZNF552	AC110597.1
PPFIA4	AC110597.1
ZNF681	AC110597.1
PCSK6	AC110597.1
ZNF239	AC110597.1
SHMT2	AC110597.1
RCC1	AC110597.1
CDT1	TRHDE-AS1
SERPINE2	TRHDE-AS1
TMEM97	TRHDE-AS1
KCNB1	AP000439.2
RNF150	AP000439.2

SNCG	AP000439.2
SPRY2	AP000439.2
ADAMTS5	AP000439.2
CHL1	AP000439.2
CLIP4	AP000439.2
PLA2R1	AP000439.2
PLEKHM3	AP000439.2
QKI	AP000439.2
LDHB	AL139220.2
DIAPH2	AL139220.2
AKR1C1	AL133373.2
KLF9	HOXC-AS3
PRCD	LINC00473
COLEC12	LINC00473
WASF3	AP005121.1
LDHB	AP005121.1
PAX9	LINC01230
TMC5	LINC01230
ZG16B	LINC01230
CAPS	LINC01230
GINS2	LINC01230
PASK	LINC01230
SDC1	LINC01230
MDK	LINC01230
MSH6	LINC01230
CORO2A	LINC01230
FANCD2	LINC01230
NAT8L	AL356740.2
TACR1	AL139412.1
STBD1	AC106028.2
ETFB	AC106028.2
MYBL2	AP001528.2
ORC1	AP001528.2
MUC5B	AP001528.2
MEX3A	AP001528.2
CDT1	AP001528.2
PAX9	AP001528.2
TMC5	AP001528.2
PAFAH1B3	AP001528.2
HIST1H2BG	AP001528.2
HIST1H2AK	AP001528.2
MDK	AP001528.2
HELLS	AP001528.2
SLC37A1	AP001528.2
RAI14	VLDLR-AS1
CFD	AL354707.1
CD300LG	AL354707.1
ALDH1L1	AL354707.1
ITIH5	AL354707.1
RDH5	AL354707.1
MYZAP	AL354707.1

PQLC2L	AL354707.1
CACNA2D1	AL354707.1
BTNL9	AL354707.1
CAVIN2	AL354707.1
SAA2	AL354707.1
KLF15	AL354707.1
TNS1	AL354707.1
DDR2	AL354707.1
MCAM	AL354707.1
FIGN	AL354707.1
DENND2A	AL354707.1
MT1X	AL354707.1
GSN	AL354707.1
DEPP1	AL354707.1
PALMD	AL354707.1
SCN9A	AL354707.1
RSPO3	AL354707.1
CRYAB	AL354707.1
CKMT2	AL354707.1
KBTBD11	AL354707.1
DSEL	AL354707.1
RGCC	AL354707.1
CHST3	AL354707.1
GRIN2B	AL354707.1
TENM1	AL354707.1
CAVIN1	AL354707.1
PELI2	AL354707.1
PEAR1	AL354707.1
NMT2	AL354707.1
TGFBR2	AL354707.1
MOB3B	AL354707.1
FAM171A1	AL354707.1
GNAL	AL354707.1
PDGFD	AL354707.1
STAT5A	AL354707.1
IRS2	AL354707.1
KAT2B	AL354707.1
SATB1	AL354707.1
CDC42BPA	AL354707.1
PLSCR4	AL354707.1
STXBP1	AL354707.1
SH3D19	AL354707.1
CNKSR2	AL354707.1
EZH1	AL354707.1
CREBRF	AL354707.1
ZDHHC2	AL354707.1
FOXO1	AL354707.1
NR1D2	AL354707.1
ADRB1	MRPS30-DT
VIT	MRPS30-DT
HJURP	AC020907.2

STIL	AC020907.2
TPRN	AC020907.2
MTURN	AC074135.1
RELN	AC074135.1
PPP1R15A	AC074135.1
CSRP2	AC074135.1
TMEM64	AC074135.1
RPS6KA3	AC074135.1
SOD2	AC074135.1
ADRA1A	AC068669.1
HS6ST3	LINC01140
CD24	LINC01140
MEX3A	LINC01140
PAX9	LINC01140
MFSD2A	LINC01140
SAPCD2	LINC01140
RAPGEFL1	LINC01140
HIST1H2BG	LINC01140
TMEM132A	LINC01140
DHRS13	LINC01140
HELLS	LINC01140
SLC37A1	LINC01140
HIST1H3G	AP001528.3
HIST1H2AI	AP001528.3
HIST1H3F	AP001528.3
BRIP1	AP001528.3
PPFIA4	AP001528.3
DNAH14	AP001528.3
SERPINE2	AP001528.3
PAFAH1B3	AP001528.3
RGL3	AP001528.3
DNAAF1	LINC01239
AGR2	LINC01239
B4GALNT4	LINC01239
C4B	LINC01239
C4A	LINC01239
DNAH5	LINC01239
MUC1	LINC01239
UBXN10	LINC01239
DNAH7	LINC01239
HIST1H2BM	AC134043.2
CDT1	AC134043.2
DNAH14	AC134043.2
SERPINE2	AC134043.2
PAFAH1B3	AC134043.2
HIST1H1D	AC134043.2
TMEM97	AC134043.2
HIST1H2AE	AC134043.2
HIST1H2BG	AC134043.2
HELLS	AC134043.2
C19orf48	AC093278.2

CLEC14A	EIPR1-IT1
ERG	EIPR1-IT1
SHTN1	AC024909.2
TMEM100	AC144450.1
P2RY12	AC144450.1
LRRN4CL	AC144450.1
DSEL	AC144450.1
CD248	AC144450.1
EFEMP1	AC144450.1
ADRB2	AC144450.1
CCND2	AC144450.1
ITPRIP	AC144450.1
C19orf48	LINC00598
CYB561	LINC00598
MYC	AC079310.1
TWIST2	AC159540.2
C19orf12	AC159540.2
KLF4	AC159540.2
MEOX1	AC159540.2
TENM1	AC159540.2
CAVIN1	AC159540.2
TMTC1	AC159540.2
STARD9	AC159540.2
PROS1	AC159540.2
CDH23	AC159540.2
ADAMTS1	AC159540.2
CDC42BPA	AC159540.2
SYNE1	AC159540.2
CNKSR2	AC159540.2
PDE8B	AC013652.1
VLDLR	AC013652.1
RCC1	AC011379.1
LY6E	LINC01695
WDR62	AL356489.2
DNAJA4	AL356489.2
CNTFR	AC092919.2
TIMP4	AP000526.1
TNMD	AP000526.1
ADH1B	AP000526.1
DPT	AP000526.1
PCDH9	AP000526.1
CLEC3B	AP000526.1
CMA1	AP000526.1
GPX3	AP000526.1
DNASE1L3	AP000526.1
EBF1	AP000526.1
ALDH1A1	AP000526.1
NRN1	AP000526.1
VWF	AP000526.1
TXNIP	AP000526.1
JDP2	AP000526.1

F8	AP000526.1
KLF2	AP000526.1
CD302	AP000526.1
FAM83D	AC026461.1
HIST1H2AI	AC026461.1
MYBL2	AC026461.1
HIST1H2AL	AC026461.1
CDC45	AC026461.1
ORC1	AC026461.1
HPX	AC026461.1
BUB1	AC026461.1
KIFC1	AC026461.1
STIL	AC026461.1
KIF23	AC026461.1
FBN2	AC026461.1
HIST1H2BE	AC026461.1
BRIP1	AC026461.1
ANO7	AC026461.1
AUNIP	AC026461.1
WDR62	AC026461.1
DNAH14	AC026461.1
SERPINE2	AC026461.1
MCM4	AC026461.1
TMEM241	AC026461.1
KDM5B	AC026461.1
SHMT2	AC026461.1
FLVCR1	AC026461.1
NEMP1	AC026461.1
COL10A1	FGF14-AS2
C3orf67	FGF14-AS2
ATG9B	FGF14-AS2
FREM1	AC141930.1
COLEC12	AC141930.1
MYC	AC141930.1
SLC30A8	AC007036.3
DNAAF1	AC007036.3
B4GALNT4	AC007036.3
DNAH7	AC007036.3
VEGFB	DGUOK-AS1
SNTA1	DGUOK-AS1
NEK7	DGUOK-AS1
TNIP1	DGUOK-AS1
ANXA5	DGUOK-AS1
MYO1C	DGUOK-AS1
PKMYT1	LIPE-AS1
HIST1H2BH	LIPE-AS1
ZNF726	LIPE-AS1
TNR	EMX2OS
PRR26	AC106820.2
MTFR2	AC132217.1
LIPG	AC132217.1

SELENOI	AC132217.1
FAM83D	AC008622.2
RAD51	AC008622.2
TNFRSF9	AC008622.2
FAM83D	AP001816.1
CDKN3	AP001816.1
CLSPN	AP001816.1
TICRR	AP001816.1
CKAP2L	AP001816.1
MELK	AP001816.1
DIAPH3	AP001816.1
PLA2G2D	AP001816.1
TNFRSF9	AP001816.1
STK36	AP001816.1
P4HA3	LINC01550
DIO2	LINC01550
C3orf67	LINC01550
ATG9B	LINC01550
STK36	LINC01550
ABCA8	AL645608.3
PALM	AL645608.3
MEOX2	AL645608.3
COLEC12	AL645608.3
TNS2	AL645608.3
CAVIN2	HELLPAR
GHR	HELLPAR
HSPB6	HELLPAR
MYOM1	HELLPAR
SCN9A	HELLPAR
CRYAB	HELLPAR
LRRN4CL	HELLPAR
NLGN1	HELLPAR
FAM107A	HELLPAR
DSEL	HELLPAR
CAVIN1	HELLPAR
TMTC1	HELLPAR
EHD2	HELLPAR
SOX5	HELLPAR
NDN	HELLPAR
GNAL	HELLPAR
PDZRN3	HELLPAR
ATF3	HELLPAR
IRS2	HELLPAR
RBMS3	HELLPAR
ADAMTS1	HELLPAR
CDC42BPA	HELLPAR
PLSCR4	HELLPAR
PLPP1	HELLPAR
SMOC1	GSEC
PQLC2L	TERC
CXCL2	TERC

FAM13A	TERC
CYYR1	TERC
MAMDC2	TERC
MOCS1	TERC
UST	TERC
TMEM250	TERC
NAV3	TERC
TBX15	AC005162.3
ABCA6	AC005162.3
CLEC14A	AC005162.3
GNG11	AC005162.3
TIE1	AC005162.3
CXCL12	AC005162.3
ZEB2	AC005162.3
CKAP2L	MANEA-AS1
MELK	MANEA-AS1
CDCA8	MANEA-AS1
PLA2G2D	MANEA-AS1
TNFRSF9	MANEA-AS1
CHAD	AC245452.1
FAM83D	AC048382.5
ORC1	AC048382.5
HPX	AC048382.5
BUB1	AC048382.5
STIL	AC048382.5
HIST1H2BE	AC048382.5
DNAH14	AC048382.5
KIAA1549	AC048382.5
RTKN2	AC048382.5
MCM4	AC048382.5
MCM8	AC048382.5
TMEM241	AC048382.5
KDM5B	AC048382.5
SHMT2	AC048382.5
FLVCR1	AC048382.5
NEMP1	AC048382.5
ADSS	AC048382.5
CKAP4	AC011247.1
ORC1	AC020916.1
MTFR2	AC020916.1
MUC5B	AC020916.1
DIAPH3	AC020916.1
LIPG	AC020916.1
TUSC5	AC005921.3
NNAT	AC005921.3
CIDEA	AC005921.3
HEPACAM	AC005921.3
SLC19A3	AC005921.3
FHL1	AC005921.3
TRHDE	AC005921.3
GHR	AC005921.3

PLAC9	AC005921.3
CLMP	AC005921.3
COX7A1	AC005921.3
RARRES2	AC005921.3
SVEP1	AC005921.3
SCN9A	AC005921.3
HOXA10	AC005921.3
SYNPO	AC005921.3
EHD2	AC005921.3
DHRS3	AC005921.3
NDN	AC005921.3
ANGPTL2	AC005921.3
FADS3	AC005921.3
PTPRM	AC005921.3
PLPP1	AC005921.3
PCBP3	LINC01814
CXCL11	LINC01619
CFAP221	LINC01619
TNFRSF9	LINC01619
JPT1	ZNF667-AS1
FAM83D	AL662844.4
HIST2H3C	AL662844.4
HIST1H3G	AL662844.4
HIST1H2AJ	AL662844.4
HIST2H3A	AL662844.4
CEP55	AL662844.4
TOP2A	AL662844.4
HIST1H1B	AL662844.4
HIST1H2AI	AL662844.4
MYBL2	AL662844.4
DTL	AL662844.4
TICRR	AL662844.4
RAD54L	AL662844.4
E2F8	AL662844.4
CDC45	AL662844.4
HIST1H2AG	AL662844.4
HIST1H2BL	AL662844.4
PRC1	AL662844.4
HIST1H3H	AL662844.4
KIF23	AL662844.4
XRCC2	AL662844.4
BRIP1	AL662844.4
SLC6A9	AL662844.4
PLA2G2D	AL662844.4
AUNIP	AL662844.4
CENPK	AL662844.4
HIST1H2BI	AL662844.4
HIST1H2BC	MIR222HG
PBK	MIR222HG
CDC20	MIR222HG
DEPDC1	MIR222HG

IQGAP3	MIR222HG
HJURP	MIR222HG
HIST1H2AL	MIR222HG
CENPF	MIR222HG
UHRF1	MIR222HG
CDK1	MIR222HG
HIST1H3F	MIR222HG
CDC45	MIR222HG
MELK	MIR222HG
HIST1H2AG	MIR222HG
CDC6	MIR222HG
KIFC1	MIR222HG
CDCA8	MIR222HG
PRC1	MIR222HG
HIST1H3H	MIR222HG
KIF23	MIR222HG
FBN2	MIR222HG
AUNIP	MIR222HG
HIST1H3D	MIR222HG
ECT2	MIR222HG
PLK1	MIR222HG
RAD51AP1	MIR222HG
AC091057.	MIR222HG
CCNF	MIR222HG
NEK2	RAB11B-AS1
HIST1H3G	RAB11B-AS1
TOP2A	RAB11B-AS1
HIST1H3F	RAB11B-AS1
HIST1H3I	RAB11B-AS1
KIF20A	RAB11B-AS1
RECQL4	RAB11B-AS1
PYCR1	RAB11B-AS1
C3orf67	RAB11B-AS1
KIF24	RAB11B-AS1
CMA1	AC092910.3
DMGDH	AC092910.3
DMRT2	AC092910.3
SCN4A	AC092910.3
